# Progress of Medical Science in Germany

**Published:** 1875-02-15

**Authors:** Edmund J. Doering


### PROGRESS OF MEDICAL SCIENCE IN GERMANY.

By Edmund J. Doering, M.D.

### LITHOTRIPSY.

THE following rules for practice in lithotripsy are laid down by Dr. Ivanchich of Vienna, who has performed this operation several hundred times during a period of more than thirty years:

1. In children under the age of twelve years, lithotripsy is admissable only in very rare cases.

2. Lithotripsy is applicable to both sexes alike.

3. After the age of twelve, the older the person the more successful the operation; in old age, especially, the success of this operation is so great that lithotripsy ought always to be considered the rule, lithotomy the exception.

4. A healthy condition of the genito-urinary organs greatly favors the success of lithotripsy; but even organic diseases of these organs, of minor severity, do not absolutely contra-indicate this operation. Thus, phimosis can be relieved by circumcision; stricture of the urethra can be removed by proper preliminary measures. Hypertrophy of the prostate gland, especially if the middle lobe is enlarged, often presents a serious obstacle, but does not necessarily prevent the performance of this operation. Whenever the spontaneous expulsion of the fragments of the calculus is prevented by the hypertrophy of the prostate gland, or by atony of the bladder, we may then accomplish this by artificial extraction. Symptomatic and even severe vesical catarrh, whether the urine reacts acid or alkaline, does not contra-indicate lithotripsy. Extreme sensitiveness of the urethra is overcome by the inhalation of chloroform. However, serious organic diseases of the kidneys, ureters or bladder absolutely contra-indicate lithotripsy, but unfortunately do not add any to the chances afforded by lithotomy.

5. Lithotripsy is most successful if there are not more than one or two calculi, small in size, not larger than a walnut, and not very hard. Calculi fully as large as a hen's egg have been successfully removed by lithotripsy, but, as a rule, render this operation difficult of execution. The chemical composition of the stone is immaterial, even if it be composed of oxalate of lime. Encysted calculi, likewise such as are situated in the neck of the bladder, and cannot be pushed back, must be removed by lithotomy. Also, if the nucleus of a vesical calculus is composed of a foreign body of great size and hardness, lithotomy must be performed.—*Allg. Wiener Med. Zeitung*, No. 1, 1875.

### TREATMENT OF PSORIASIS.

Dr. Zimmerhaus reports the very best results from the administration of carbolic acid in the two following cases of psoriasis:

1. M. D., 26 years of age, has been suffering for several years with psoriasis, for which he has been taking numerous remedies, especially Fowler's solution and the bichloride of mercury, but without deriving any benefit therefrom. On inspection the author found on the right and a portion of the left arm numerous irregularly formed patches covered with dry, white scales. Carbolic acid was prescribed in pill form, giving at first six grains during the day, and gradually increasing the dose to sixteen grains pro die. In fourteen days the scales had disappeared and red spots were seen in place of the patches. The dose of the carbolic acid was then diminished to four grains daily and cod-liver oil applied locally, till finally, in all five weeks after the commencement of the treatment, the patient was discharged cured. As late as three months after his dismissal no relapse had occurred.

2. M. B., a young girl thirteen years of age, under treatment for an affection of the eyes. The author's attention being called to an eruption on the skin, he discovered numerous small infiltrated patches, covered with dry scales, scattered over the back and breech of the child, presenting the usual characteristic appearances of psoriasis. The same treatment was adopted as in the former case, the dose of the carbolic acid being regulated according to the age of the child. In four weeks not a trace of the eruption could be discovered. Five months have elapsed since, but as yet the eruption has not reappeared.—*Wiener Med. Presse*, No. 42, 1874.

ADDISON'S DISEASE.

Dr. Verardini has just published a memoir on the above, embracing an analysis of all cases of Addison's disease published during the last fifteen years, the conclusions of which are summed up thus:

1. A bronze-like discoloration of the skin, without a diseased condition of the supra-renal capsules, is often observed in tuberculosis. On the other hand the supra-renal capsules are frequently found tuberculous, and having undergone caseous degeneration, without the peculiar bronzing of the skin appearing during life.

2. We find in various diseases anæmia and bronzing of the skin associated without being compelled to consider this a separate type of disease.

3. In connection with the above, loss of muscular and nervous power are often found, and yet no disease of the supra-renal capsules can be demonstrated after death.

These conclusions are in conformity with the theory advanced by the author so early as 1858, in a publication in which he states that Addison's disease must be looked upon as a cachexia, arising from various causes, and not depending solely on an affection of the supra-renal capsules.—*Allg. Med. Central Zeitung*, No. 102.

